# Bone marrow involvement by classic Hodgkin lymphoma

**DOI:** 10.1002/jha2.391

**Published:** 2022-01-24

**Authors:** Mohammad Barouqa, April Chiu

**Affiliations:** ^1^ Mayo Clinic Rochester Minnesota USA

A 70‐year‐old male patient with a history of prostate cancer (Gleason score 7) treated with proton beam and Leuprolide presented with fatigue, fever, and new cytopenia (Hgb: 10.5 g/dL, RBC: 3.57 × 10 ^12^/L, MCV: 94.1 fL, RDW: 17.8%, WBC: 5.4 × 10 ^9^, Platelet: 104 × 10 ^9^/L). CT scan showed periportal and retroperitoneal lymphadenopathy while positron emission tomography‐computed tomographic scan showed asymmetric increased fluorodeoxyglucose uptake in the bone marrow. Review of the peripheral blood smear did not show any remarkable findings; however, the bone marrow aspirate revealed the presence of large atypical lymphoid cells with reticulated chromatin and prominent nucleoli (Top left, X100). Few cells were bilobed with prominent nucleoli and ample amount of cytoplasm (Top middle, ×100). The core biopsy revealed an atypical lymphohistiocytic infiltrate composed of large, atypical cells with irregular nuclear contours, prominent nucleoli, and abundant amount of cytoplasm, consistent with Reed–Sternberg cells (RS) and variants (Top right, ×60). These atypical cells were surrounded by a mixed inflammatory cell infiltrate composed of small lymphocytes, histiocytes, and granulocytes (neutrophils and eosinophils); which altogether occupied 60% of the bone marrow cellularity rendering the bone marrow hypercellular with decreased but morphologically unremarkable trilineage hematopoiesis. Background fibrosis was not seen. CD15 stained the RS cells and granulocytes (neutrophils and eosinophils) (Bottom left, black arrow, X100). CD30 highlighted the RS cells (Bottom middle, black arrow, X100). PAX 5 was weakly expressed in the RS cells (Bottom right, black arrow, X100). Subsequent excisional biopsy of the retroperitoneal nodules confirmed the diagnosis.


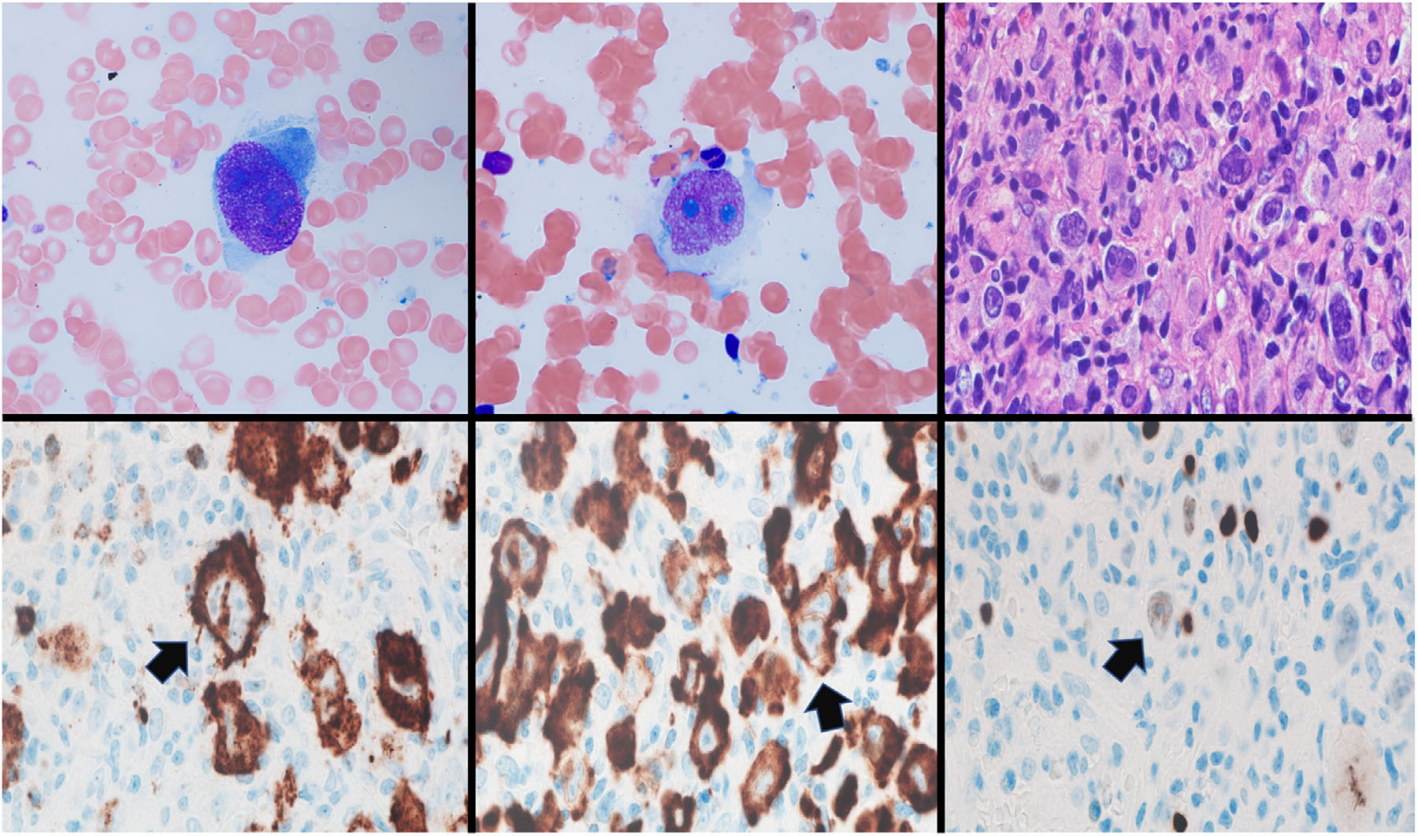


The findings in this case are consistent with classic Hodgkin lymphoma involving the bone marrow. The presence of RS cells and their variants in the bone marrow aspirates is an important clue that, while RS cells are uncommon in aspirates, they play an important role in the evaluation and staging of the disease required to provide an accurate diagnosis, prognosis, and the best treatment for the patients.

## CONFLICT OF INTEREST

The authors have declared no conflict of interest.

